# Metabolic risk factors, physical activity and physical fitness in azorean adolescents: a cross-sectional study

**DOI:** 10.1186/1471-2458-11-214

**Published:** 2011-04-06

**Authors:** Carla Moreira, Rute Santos, José Cazuza de Farias, Susana Vale, Paula C Santos, Luísa Soares-Miranda, Ana I Marques, Jorge Mota

**Affiliations:** 1 Research Centre in Physical Activity, Health and Leisure, Faculty of Sport, University of Porto, Portugal; 2 Federal University of Paraiba, Physical Education Department, Brazil; 3 Department of Physiotherapy, School of Health Technology of Porto, IPP, Portugal

## Abstract

**Background:**

The prevalence of metabolic syndrome has increased over the last few decades in adolescents and has become an important health challenge worldwide. This study analyzed the relationships between metabolic risk factors (MRF) and physical activity (PA) and physical fitness (PF) in a sample of Azorean adolescents.

**Methods:**

A cross-sectional school-based study was conducted on 417 adolescents (243 girls) aged 15-18 from the Azorean Islands, Portugal. Height, weight, waist circumference, fasting glucose, HDL-cholesterol, triglycerides, and blood pressure were measured. A sum of MRF was computed, and adolescents were classified into three groups: no MRF, one MRF and two or more MRF. PA was assessed by a sealed pedometer. PF was assessed using five tests from the Fitnessgram Test Battery. Dietary intake was obtained using a semi-quantitative food frequency questionnaire.

**Results:**

Mean daily steps for girls and boys were 7427 ± 2725 and 7916 ± 3936, respectively. Fifty-nine percent of the adolescents showed at least one MRF and 57.6% were under the healthy zone in the 20 m Shuttle Run Test. Ordinal logistic regression analysis showed that after adjusting for sex, body mass index, socio-economic status and adherence to a Mediterranean diet, adolescents who were in the highest quartile of the pedometer step/counts (≥9423 steps/day) and those who achieved the healthy zone in five tests were less likely to have one or more MRF (OR = 0.56;95%CI:0.33-0.95; OR = 0.55;95%CI:0.31-0.98, respectively).

**Conclusions:**

Daily step counts and PF levels were negatively associated with having one or more MRF among Azorean adolescents. Our findings emphasize the importance of promoting and increasing regular PA and PF to reduce the public health burden of chronic diseases associated with a sedentary lifestyle.

## Background

Metabolic Syndrome (MetS) comprises a major risk for chronic disease and is rapidly increasing in prevalence worldwide in association with rising childhood obesity and a sedentary lifestyle [1]. Emerging evidence suggests that children who have clustered MRF are at increased risk for developing cardiovascular diseases and type II diabetes in adulthood [2]. The most recognized cardiovascular disease risk factors are HDL-cholesterol, total cholesterol, triglycerides, total and central body fat, insulin resistance, blood pressure, and cardiorespiratory fitness (CRF). The prevalence of adolescents who are physically inactive, in conjunction with the rising prevalence of obesity, are a major threat to health in the twenty-first century [3]. Thus, adolescence is a critical period, because this is when the individual takes control of his/her lifestyle. It is during this period that engagement in PA might contribute to a physically active lifestyle that lasts into adulthood [4].

Regular PA is reported to be a protective factor against several diseases, such as obesity, hypertension, type II diabetes, [3] and MetS [5]. The use of pedometers to quantify PA has led to the development of guidelines for recommended steps/day for children (aged 6-12). Recommendations range from 11,000 to 15,000 steps/day for girls and boys, respectively [6,7]. However, there are no internationally accepted pedometer cut-points for adolescents.

Studies examining the association between CRF and clustered metabolic risk in youth report an inverse relationship, indicating that as the CRF increases, the risk of an unfavorable metabolic risk profile is reduced [8]. Indeed, both lower levels of PA and CRF have been associated with a higher clustering of MRF in young people [9]. Evidence also suggests that sedentary behavior and low levels of PA and CRF in youth continue into adulthood and may predispose young people to disease later in life [10].

Associations between increased CRF and several MRF have been repeatedly found. However, the information about the relationship between the overall PF levels with MRF is scarce. To our knowledge, there is only one study that has compared coronary heart disease risk factors to PA measured objectively by pedometry [11], and, to date, there are no studies that have analyzed the relations between MRF and PA (measured with pedometers) and overall PF levels in adolescents. The present study fills this gap by analyzing these relationships in a sample of Azorean adolescents.

## Methods

### Study design and sampling

Data for the present study derived from a longitudinal school-based study, the Azorean Physical Activity and Health Study II, aimed to evaluate PA, PF, overweight/obesity prevalence, health related quality of life and related factors beginning in 2008. Study design and sampling are reported elsewhere [12]. For this study we only considered the 517 adolescents with metabolic data evaluated in 2009; of this total, 100 did not have valid pedometer information as described below in the PA section. Therefore, the final sample included in this cross-sectional analysis was comprised of 417 adolescents (243 girls and 174 boys) aged 15 to 18 (mean age 16.5 ± 0.9).

All participants in this study were informed about the objectives of the work, and the parent or guardian of each participant provided written informed consent. The study was approved by the faculty and the Portuguese Foundation for Science and Technology ethics committee and conducted in accordance with the Declaration of Helsinki for Human Studies of the World Medical Association.

### Anthropometric Measures

Height was measured to the nearest millimeter in bare or stocking feet with the adolescent standing upright against a stadiometer (Holtain Ltd., Crymmych, Pembrokeshire, UK). Weight was measured to the nearest 0.10 kg, lightly dressed using a portable electronic weight scale (Tanita Inner Scan BC 532). Body mass index (BMI) was calculated as weight/height squared (kg/m^2^) and used to define thinness, normal weight, overweight and obese group [13,14]. However, for analytical purposes the thinness and normal weight as well as overweight and obese adolescents were collapsed in two groups, thinness/normal weight and the overweight/obese, respectively. Waist circumference (WC) was measured twice with a metal anthropometric tape midway between the lower rib margin and the iliac crest at the end of normal expiration [15] and the average of the two measures was used for analysis.

### Blood Pressure

Blood pressure (BP) was measured using the Dinamap adult/pediatric vital signs monitors, model BP 8800 (Critikon, Inc., Tampa, Florida). Measurements were taken by nurses, all adolescents required to sit and rest for at least five minutes prior to the BP test. The participants were in a seated, relaxed position with their feet resting flat on the ground. Two measurements in the right arm were taken, after five and ten minutes of rest. The mean of these two measurements was considered. If the two measurements differed by 2 mmHg or more, a third measure was taken.

### Blood Sampling

Blood samples were collected from the antecubital vein between 8:00 and 10:00 a.m. in a sitting position after ten hours of fasting. The blood samples were drawn in vacuum tubes gel (Sarstedt) in order to obtain values of plasmatic high-density lipoprotein cholesterol (HDL-cholesterol), triglycerides (TG) and glucose. After resting at room temperature for about 30 minutes, the samples were centrifuged for 10 minutes at 3000 rpm to obtain serum. Samples were divided into aliquots, separated within 30 minutes, and stored at -80°C until analyzed. The following analyses were measured on a Cobas Integra 400 Plus, (ROCHE): HDL-cholesterol using the precipitation of the Apolipoprotein B containing lipoproteins with dextran-magnesium-chloride; TG using the Glycerokinase-Glycerolphosphateoxidase-Peroxidase (GK-GPO-POD) method; and glucose using the hexokinase method. The biochemical evaluation of all participants from the different islands was conducted in the same laboratory.

### Metabolic Risk Factors

Since there is no consensus regarding the establishment of a universal criterion for the definition of MetS in children and adolescents, it was decided to compute a sum of MRF based on five cut-off points of the MetS International Diabetes Federation criteria [16] using the following measurements: WC above the 90th percentile for age and sex, for adolescents aged <16 (or adult criteria if the 90th percentile is lower) and for adolescents aged ≥16 (>80 cm for girls and >94 cm for boys); TG≥150 mg/dL; HDL-cholesterol<40 mg/dL for both genders except HDL-cholesterol<50 mg/dL for girls aged ≥16 years; fasting glucose ≥100 mg/dL; and systolic BP≥130 mmHg or diastolic BP≥85 mmHg. For adolescents aged<16, the WC percentiles were used (sex and age-specific 90th percentile) for British children [17] as they had been previously used by Ekelund [18] for Portuguese adolescents. Adolescents were then classified in three groups: no MRF, one MRF and two or more MRF.

### Socio-Economic Status

The highest level of parental education (in completed years of education) was considered as a proxy of socio-economic status. Similar procedures had previously been applied in the Portuguese context [19].

### Mediterranean Diet Score

Dietary intake was obtained using a semi-quantitative food frequency questionnaire regarding the previous 12 months comprised of 82 food and beverage items [20]. A Mediterranean diet score was adapted from the alternate Mediterranean score [21]. This was constructed based on the intake of group foods (pulses, vegetables, fresh fruits, nuts, whole grains, fish, red and processed meats, ethanol) as well as on the ratio of monounsaturated to saturated fat. Using the sex-specific median of the study's participants as a cut-off value for each of the components, 1 point was given when intake ≥ median and 0 points for intakes < median for all items except for red and processed meats and ethanol (below median = 1 point). If participants met all the characteristics of the Mediterranean diet, they achieved the highest score possible (nine points), reflecting maximum adherence. If they met none of the characteristics, the score was minimum (zero), reflecting no adherence at all. Based on these results, participants were categorized into tertiles (low, medium, and high).

### Physical Activity

Physical activity (PA) was assessed objectively using a sealed pedometer (Kenz Lifecorder Plus, Suzuken Co. Ltd, Nagoya, Japan) worn over seven consecutive days. Participants with fewer than three days (two weekdays and one weekend day) of activity recorded were eliminated from the data analysis (74 adolescents) in accordance with previous findings [22]. Another 26 adolescents whose leisure time PA was swimming were also deleted from the analysis, because the pedometer is not capable of detecting water activities. Adolescents were familiarized with the pedometers during a physical education class before the monitoring period. On the first day of monitoring (Monday), adolescents were instructed on pedometer attachment (at the waist), its removal (only during showering, bathing, swimming, or sleeping), and re-attachment each morning before going to school. Pedometer values were taken as the average number of steps/day and weighted according to the ratio of weekdays to weekends. Mean step counts were divided into age-adjusted quartiles.

### Physical Fitness

Several studies have shown that in adolescence low CRF is a predictor of cardiovascular disease risk factors [8,23,24]; and therefore, reaching the HZ in the 20mSRT was a mandatory criterion to evaluate the PF levels. Health-related components of PF were evaluated using the Fitnessgram Test Battery 8.0. The Fitnessgram is included in physical education curricula, and the five tests recommended in the Portuguese National Program were used in this study. These activities include curl-ups, push-ups, trunk lifts, a 20 m shuttle run test (20mSRT), and the modified-back-saver-sit-and-reach. The 20mSRT was used to evaluate CFR. The curl-up, push-up and trunk lift tests were used to evaluate strength. The modified-back-saver-sit-and-reach test was used to evaluate flexibility. All tests were conducted according to the Fitnessgram measurement procedures [25]. According to the results of each test, adolescents were classified in two groups according to their ages and sex-specific cut-off points of the Fitnessgram 8.0 criteria, as belonging to the healthy zone (HZ) or above or under the healthy zone (UHZ). Adolescents were classified in different groups: UHZ in the 20mSRT, HZ or above in the 20mSRT plus one test, and continuing consecutively through plus four tests.

### Statistical Analysis

Descriptive data are presented as means and standard deviation unless otherwise stated. All variables were checked for normality and appropriately transformed if necessary. The HDL-C, TG, systolic BP, and WC were logarithmically transformed. Independent sample *t *tests with Bonferroni corrections were performed to compare sexes in continuous variables.

Ordinal logistic regression was used to verify the relationship between MRF (dependent variable in ordinal scale, and it was coded as 0 = no MRF; 1 = one MRF; and 2 = two or more MRF), with PA, and overall PF levels (independent variables). Two models were created for analysis. The first model analyzed the relationship between the MRF and PA, and the second model analyzed the relationship between MRF and overall PF levels. Both models were adjusted for sex, BMI, socio-economic status and adherence to the Mediterranean diet. The assumption of proportional odds for both models was examined using the Brant test (first model: χ^2 ^= 5.72, p = 0.573; second model: χ^2 ^= 8.10, p = 0.151). Data were analyzed using the Stata 10.0 software (Stata Corp., College Station, U.S.) and SPSS for Windows (version 17.0). A p value under 0.05 denoted statistical significance.

## Results

Descriptive characteristics of the adolescents are shown in Table 1. Boys had higher levels of height, weight, WC, systolic BP and glucose than girls (p < 0.001 for all), whereas girls had higher values of HDL-C (p < 0.001). No differences in age, BMI, diastolic BP, TG and mean step counts were observed between the sexes (p > 0.05). In the overall sample 41.0% had no MRF, 40.3% had one MRF, and 18.7% had two or more MRF. The prevalence of adolescents UHZ in the 20mSRT was 57.6% (72.0% for girls and 37.4% for boys, p < 0.001). In the sample 17.5% were categorized in the HZ in five tests (girls 9.1% and boys 29.3%, p < 0.001).

**Table 1:** Descriptive characteristics of the study sample.

Variables	Total (n = 417)	Girls (n = 243)	Boys (n = 174)
Age, years	16.5 ± 0.9	16.5 ± 1.0	16.4 ± 0.8
Height, cm	165.0 ± 13.6	160.0 ± 11.0	172.0 ± 1.0*
Weight, kg	63.1 ± 12.5	58.6 ± 10.0	69.3 ± 13.1*
BMI, kg/m^2^	22.9 ± 3.7	22.7 ± 3.5	22.7 ± 3.5
Waist circumference, cm	79.3 ± 10.7	78.0 ± 10.3	81.2 ± 11.1*
Systolic BP, mm Hg	115.2 ± 15.3	111.8 ± 13.8	120.0 ± 16.1*
Diastolic BP, mm Hg	66.4 ± 9.4	66.3 ± 10.0	66.5 ± 8.6
HDL-cholesterol, mg/dl	55.6 ± 13.4	59.3 ± 13.0	50.5 ± 12.3*
Triglycerides, mg/dl	70.7 ± 35.1	72.5 ± 34.6	68.1 ± 35.6
Glucose, mg/dl	86.3 ± 8.8	84.5 ± 8.8	88.9 ± 8.3*
Mean step counts	7631 ± 3289	7427 ± 2725	7916 ± 3936
**Number of MRF (%, n)**			
No risk factors	41.0 (171)	35.8 (87)	48.3 (84)
1 risk factor	40.3 (168)	42.0 (102)	37.9 (66)
2 or more risk factors	18.7 (78)	22.2 (54)	13.8 (24)
**Physical Fitness (%, n)**			
UHZ 20mSRT	57.6 (240)	72.0 (175)	37.4 (65)
HZ 20mSRT + HZ in 1 test	1.7 (7)	1.6 (4)	1.7 (3)
HZ 20mSRT + HZ in 2 tests	7.7 (32)	6.2 (15)	9.8 (17)
HZ 20mSRT +HZ in 3 tests	15.6 (65)	11.1 (27)	21.8 (38)
HZ 20mSRT + HZ in 4 tests	17.5 (73)	9.1 (22)	29.3 (51)

Values are means (SD). Student's *t *test, with Bonferroni corrections for differences between sex: *p < 0.001; BMI, body mass index; BP, blood pressure; HDL, high-density lipoprotein; MRF, metabolic risk factors; UHZ, under healthy zone; HZ, healthy zone; 20mSRT, 20 m Shuttle Run Test.

Results of the ordinal logistic regression analysis for MRF and PA, adjusted for sex, BMI, socio-economic status and adherence to a Mediterranean diet, are shown in Table 2. Adolescents who were in the highest quartile of the pedometer step counts were less likely to have one or more MRF compared to those in the lowest quartile (OR = 0.56; 95%CI:0.33-0.95).

**Table 2:** Ordinal logistic regression showing estimating results with MRF as dependent variable and pedometer step counts into age-adjusted quartiles as independent variable.

Pedometer step quartiles	OR unadjusted (95% CI)	p	OR adjustedª (95% CI)	p
1^st^: ≤5621 steps	1		1	
2^nd^: 5622 -7403 steps	0.59 (0.35 - 0.98)	0.042	0.65 (0.38 - 1.11)	0.116
3^rd^: 7404 - 9422 steps	1.22 (0.74 - 2.02)	0.431	1.20 (0.71 - 2.02)	0.490
4^th^: ≥ 9423 steps	0.52 (0.31 - 0.87)	0.012	0.56 (0.33 - 0.95)	0.032

OR, odds ratios; CI, confidence intervals; 1, reference category; ª adjusted for sex, body mass index (categories), socio-economic status and adherence to a Mediterranean diet.

Similar results were found when an analysis was performed to assess the relationship between the MRF and the overall PF levels. Adolescents who achieved the HZ in five tests (20mSRT plus 4 tests) were less likely to have one or more MRF compared to those who were in the UHZ, after adjusting for sex, BMI, socio-economic status and adherence to a Mediterranean diet (OR = 0.55; 95%CI:0.31-0.98) (Table 3).

**Table 3:** Ordinal logistic regression showing estimating results with MRF as dependent variable and overall physical fitness levels as independent variable.

Overall Physical Fitness levels	OR unadjusted (95% CI)	p	**OR adjusted**^**a**^ (95% CI)	p
UHZ 20mSRT	1		1	
HZ 20mSRT + HZ in 1 test	0.27 (0.62-1.13)	0.072	0.46 (0.96-2.21)	0.333
HZ 20mSRT + HZ in 2 tests	0.30 (0.15-0.61)	0.001	0.48 (0.23-1.01)	0.054
HZ 20mSRT +HZ in 3 tests	0.42 (0.25-0.71)	0.001	0.73 (0.427-1.28)	0.269
HZ 20mSRT + HZ in 4 tests	0.27 (0.16-0.46)	<0.001	0.55 (0.31-0.98)	0.043

OR, odds ratios; CI, confidence intervals; 1, reference category; UHZ, under healthy zone; HZ, healthy zone; 20mSRT, 20m Shuttle Run Test; ^a^adjusted for sex, body mass index (categories), socio-economic status and adherence to a Mediterranean diet.

## Discussion

The main findings from this study indicate that adolescents who are more active (≥9423 steps/day) and those who achieve the HZ in five tests have lower odds for having one or more MRF. Other important findings of the study indicate that 59% of the participants showed at least one MRF and 57.6% were UHZ in the 20mSRT.

In this study, the mean daily step counts were 7427 for girls and 7916 for boys. These results are below the ranges of steps/day reported for adolescents by previous authors [6,26]. A three-year follow-up study of adolescents in Sweden showed that the daily mean step for boys was 11,892 and for girls 12,271 [27], while US children took between 11,000 and 13,000 steps/day [28]. The Canadian Physical Activity Levels Among Youth Study reported that Canadian children and youth (aged 5 to 19) take an average of 11,356 steps/day [7]. The ranges of steps/day reported in these studies are much higher than those found for the Azorean adolescents. Some explanations of these differences could be environmental, such as community design as well as other cultural differences.

Given the lack of recommended step counts for the adolescent population, we decided to divide the mean step counts/day into quartiles adjusted to the adolescent's age. In this study, it was observed that the value of the 4^th ^quartile was 9423 steps/day, which is close to the step count cut-off of 10,000 proposed for adults, [29] and as adolescents reach adulthood, they begin to approximate adults in PA patterns.

Though this study has not examined PA intensity, Wild et al. [30] showed that adolescents who reported meeting the recommendations for both moderate and vigorous PA accumulated the most steps/day. Moreover, the highest levels of PA were associated with healthy outcomes. In this study, the finding of very low PA patterns suggests that Azorean adolescents may be at an increased risk for obesity, hypertension, type II diabetes, and coronary heart disease [3]. We found no significant sex differences in PA, although Azorean boys have slightly higher levels of PA compared to girls, which is consistent with the findings reported by Hardman et al. [26].

Reduction in PA is linked to increases in childhood/adolescent obesity [31] and MetS [5]. In the EYHS using a cross-sectional multicenter study of 1732 children and adolescents, Andersen et al. [32] showed that the risk of having clustered risk factors decreased in a dose-gradient manner with increased moderate-to-vigorous PA. In another study with the Danish cohort of 9- to 10-year-old children, PA was also shown to be inversely associated with clustered metabolic risk [9]. However, the total volume of PA necessary for preventing cardiovascular disease risk in adolescents is not clear, and no pedometer guidelines have been set for adolescents.

The lack of PF has also been associated with the development of cardiovascular disease risk factors in youth, such as lipid disorders, high BP and insulin resistance, among others [24]. Our results showed a positive influence of overall PF levels on MRF. Adolescents who are in the HZ in five tests had lower odds of having MRF than those who were UHZ. Some studies have shown that PF levels track from adolescence to adulthood, [10] with moderate to strong coefficients for CRF and strength, respectively [33].

Ruiz et al. have also shown an inverse association between CRF and clustered MRF in 9-10-year-old Swedish and Estonian children [23]. Reinforcing this idea, Ortega et al. [24] reported that children and adolescents with higher levels of CRF also have a more favorable cardiovascular profile compared to their unfit counterparts. Conversely, some studies have shown that high levels of CRF and PA are associated with a favorable metabolic risk profile [32]. Similarly, Ekelund et al. [34] found independent inverse associations of PA and CRF with clustered metabolic risk. However, direct comparisons with our study are difficult because in this study PF was evaluated using five tests from the Fitnessgram Test Battery, while in other studies PF was measured using only the CRF level [8,23,24].

Regarding the relationship between PA and CRF levels with MRF, our study indicated that although Azorean adolescent girls had similar step counts to boys, they had lower CRF levels and had more prevalence of MRF. A partial explanation for these differences could be the fact that boys, in general, are more vigorous in PA [35,36]; therefore, this may to lead to higher CRF levels compared to girls. With this in mind, boys may be more protected in relation to MRS than girls. Although in our study we did not assess PA intensity, it is possible that boys engaged in more vigorous PA than girls, leading to high CRF levels.

The main strength of the current study is that PA was assessed objectively by pedometers, which are a valid and reliable measure. Moreover, walking is one of the most common forms of PA and is easily captured by a pedometer. Their relatively low cost and ease of administration make them attractive for use in field-based PA studies. The use of field tests for PF assessment, which can be administered in school settings where a large number of participants can be tested simultaneously, enhances participant motivation, making it a valuable tool for studying PF in youth. Another aspect to note is the specificity of the place of the study, which was conducted in the Azores Islands. Some studies have been published on Azorean adults [37-39], but in adolescence, the information is scarce [12]. This study is limited because it consisted of a cross-sectional analysis, which limits inferences about causality and its direction. Another limitation of the pedometer it is that it does not provide information about PA intensity, nor does it record activities such as bicycling, swimming and climbing. Moreover, in this study a four sex-and-age-specific BMI categories (thinness, normal weight, overweight and obese) were not analyzed since there were fewer adolescents in the limit categories (thinness and obese), however, in the thinness category none of the adolescents had one or more MRF or had low fitness levels. As evidenced by Bovet et al. [40], there was a trend toward lower performance in lean students, as compared to students with normal weight for all fitness tests.

## Conclusion

In conclusion, the results of this study indicate that adolescents who were more active (≥9423 steps/day) and those who achieved the HZ in five tests were less likely to have one or more MRF, after adjusting for sex, BMI, socio-economic status and adherence to a Mediterranean diet. Our findings emphasize the importance of promoting and increasing regular PA and PF to reduce the public health burden of chronic diseases associated with a sedentary lifestyle. Further research is warranted to quantify how much PA and PF are needed to prevent and reduce the risk in those who already have one or more MRF, highlighting potential sex and BMI differences.

## Competing interests

The authors declare that they have no competing interests.

## Authors' contributions

CM, RS and JM have made substantial contributions to conception and design of data. CM and RS and have carried out the data collection, statistical analysis, interpretation of data and wrote the manuscript. JCFJ, SV, LSM, AIM and PCS have been involved in drafting the manuscript. All authors read and approved the final manuscript.

## Pre-publication history

The pre-publication history for this paper can be accessed here:

http://www.biomedcentral.com/1471-2458/11/214/prepub
